# Endotyping of Chronic Rhinosinusitis With and Without Polyp Using Transcription Factor Analysis

**DOI:** 10.3389/fcimb.2018.00082

**Published:** 2018-03-27

**Authors:** Pongsakorn Tantilipikorn, Nitat Sookrung, Soranart Muangsomboon, Jate Lumyongsatien, Anan Bedavanija, Triphoom Suwanwech

**Affiliations:** ^1^Division of Rhinology and Allergy, Department of Otorhinolaryngology, Faculty of Medicine Siriraj Hospital, Mahidol University, Bangkok, Thailand; ^2^Office of Research and Development, Faculty of Medicine Siriraj Hospital, Mahidol University, Bangkok, Thailand; ^3^Department of Pathology, Faculty of Medicine Siriraj Hospital, Mahidol University, Bangkok, Thailand

**Keywords:** Thailand, endotyping, chronic rhinosinusitis, chronic rhinitis, polyp, transcription factor analysis

## Abstract

Inflammation of the nose and paranasal sinus or rhinosinusitis (RS) is a significant global health problem that is both very common and very costly to treat. Previous reports reveal variability in histology and mechanism of inflammation in patients with chronic rhinosinusitis with and without polyp (CRScNP and CRSsNP, respectively). There are various methods and hypothesis that try to explain this variability. Accordingly, the aim of this study was to investigate the incidence of each type of sinonasal inflammation among patients diagnosed with CRScNP or CRSsNP using transcription factor analysis (TFA). This study included mucosa specimens from nose/paranasal sinuses from patients with chronic rhinitis (CR), CRSsNP, or CRScNP that were obtained at the Department of Otorhinolaryngology, Faculty of Medicine Siriraj Hospital, Mahidol University, Bangkok, Thailand during the June 2009 to May 2012 study period. TFA was employed to measure the following transcription factors: T-box transcription factor (T-bet) for Th1, GATA binding protein 3 (GATA-3) for Th2, retinoic acid-related orphan receptor C (RORC) for Th17, and forkhead box P3 (FOXP3) for Treg. Forty-one subjects (22 males, 19 females) were enrolled, with a mean age of 45.93 ± 13 years. Twenty-six patients were diagnosed with CRScNP, 7 with CRSsNP, and 8 with CR (controls). The majority of CRScNP specimens (76.9%) had eosinophil count greater than 100 cells/high-power field (HPF). Mean eosinophil count was 930.08 ± 1,399 cells/HPF (range: 17–5,570). Th2 transcription factor (GATA-3) was statistically significantly higher in the CRScNP group than in the CRS and control groups (*p* < 0.001); whereas, Treg transcription factor (FOXP3) was statistically significantly lower in the CRScNP group than in the CRSsNP and control groups (*p* < 0.001). The transcription factors for Th1 and Th17 (T-bet and RORC, respectively) were not significantly different among the three groups. The result of transcription factor analysis revealed hyperfunction of Th2 in patients with CRScNP, which might result in hypereosinophilic infliltration in the polyps. One explanation for this finding is the decreased activity of Treg. Although environment-host interaction is the most probable hypothesis, the etiology of aberrant adaptive immunity needs to be elucidated.

## Introduction

Inflammation of the nose and paranasal sinus or rhinosinusitis (RS) is a significant global health problem that is both very common and very costly to treat. Acute rhinosinusitis (ARS) can be caused by bacterial or viral infection. It is widely accepted that the pathophysiology of RS involves, either entirely or in part, the ostiomeatal complex (OMC). Treatment for RS consists of a combination of antimicrobial therapy, drainage of sinus secretions, and treatment of underlying cause, especially allergic condition.

The etiology of chronic rhinosinusitis (CRS) is less clear. The proposed pathogenesis of chronic inflammation of nasal and sinus mucosa is thought to be multifactorial, including the presence of recalcitrant microorganism(s). Novel theories have been proposed, including biofilms and superantigens; however, dysregulation of T lymphocyte remains the dominant factor in chronic inflammation (Bachert et al., 2000; Akdis et al., 2005).

Compared to patients with chronic rhinitis (CR), CRS patients have extension of inflammatory process from the nasal cavities to the sinus mucosa. CRS can be subclassified, both clinically and immunobiologically, into CRS with nasal polyp (CRScNP or CRSwNP) and CRS without nasal polyp (CRSsNP, or CRS) (Fokkens et al., 2012). CRScNP shows predominant T helper 2 (Th2) with eosinophil inflammation, whereas CRS shows predominant Th1 inflammation (Van Zele et al., 2006; Akdis et al., 2013). Accordingly, the study of inflammatory cytokines reveals high IFN-γ and TGF-β for CRSsNP, and high IL-5 for CRScNP. The polarization of Th1 and Th2 is influenced by the function of another subgroup of T cells, which are called T regulatory (Treg) cells. Another T cell subgroup (Th17) was studied to investigate its role in CRSsNP/CRSwNP (Saitoh et al., 2010). The predominant inflammatory cytokine of Th17 is interleukin (IL)-17.

In addition to the measurement of inflammatory cytokines, evaluation of intracellular mechanism by transcription factor analysis (TFA) is an alternative method to evaluate the functions of Treg and conventional T helper (Th) cells. T-box transcription factor (T-bet), GATA binding protein 3 (GATA-3), retinoic acid-related orphan receptor C (RORC), and forkhead box P3 (FOXP3) is the transcription factor for Th1, Th2, Th17, and Treg, respectively.

Dysregulation of T cells can cause different histology of sinus/nasal mucosa. Sinus mucosa in CRScNP cases most often shows abundant eosinophils, especially in specimens from European countries (Fokkens et al., 2012). Recent data from China showed distinct immunopathologic involvement by the presence of noneosinophilic inflammation in over half of CRScNP cases (Cao et al., 2009). This finding correlates with data reported from our center in 2002, which showed 81.4% of specimens as noneosinophilic (Jareoncharsri et al., 2002).

Data that elucidates this variability in histology and type of inflammation among patients with CRS (with and without polyp) is lacking. Accordingly, the aim of this study was to investigate the incidence of each type of sinonasal inflammation among patients diagnosed with CRSsNP or CRScNP using TFA.

## Materials and methods

### Patients

This study included mucosa specimens from nose/paranasal sinuses from patients with CR, CRSsNP, or CRScNP that were prospectively obtained at the Department of Otorhinolaryngology, Faculty of Medicine Siriraj Hospital, Mahidol University, Bangkok, Thailand during the June 2009–May 2012 study period. Siriraj Hospital is Thailand's largest national tertiary referral center. The protocol for this study was approved by the Siriraj Institutional Review Board (SIRB) (COA no. Si 255/2009). This study complied with the principles set forth in the Declaration of Helsinki and all of its subsequent amendments, and written informed consent was obtained from all study participants.

In CRScNP patients, samples of polyp tissue were taken during endoscopic sinus surgery (ESS). In CRSsNP patients, samples of ethmoidal/ostiomeatal complex mucosa were obtained. Diagnosis of CRS was based on history, nasal endoscopy, and computed tomography (CT) of paranasal sinuses according to European Position Paper on Rhinosinusitus and Nasal Polyps 2007 (EPOS 2007) guidelines (Fokkens et al., 2007). CT scans were scored using the Lund-Mackay scoring system (Lund and Kennedy, 1995). Polyps were scored by size and extension (range 0–3) based on Davos classification (Lund and Kennedy, 1995).

In patients with CR, turbinate mucosa from inferior turbinate tissue that was removed during inferior turbinoplasty to treat chronic nasal obstruction was collected for use as a control specimen. Allergic status was evaluated by skin prick test or elevated serum-specific immunoglobulin E (IgE) to common inhalant allergen. Any patient with suspected asthma based on patient history was evaluated by a pulmonologist to confirm or deny the presence of asthma.

No included patient used oral or nasal steroid within 4 weeks of biopsy/surgery. Prior to surgery/biopsy, patients with acute exacerbation of rhinosinusitis or allergic rhinitis were treated until that episode subsided. Antibiotics, antihistamines, and antileukotrienes were discontinued 2 weeks before biopsy/surgery.

### Histologic study

All nasal polyp specimens were sent to the Department of Pathology, Faculty of Medicine Siriraj Hospital for histologic evaluation. Sections were stained with hematoxylin-eosin (HE). The number of eosinophils was counted using light microscopy (400x) and scored by a pathologist (SM) who was blinded to the diagnosis and clinical data. The eosinophil count was scored according to the system described by Van Zele et al. (2006). The analysis included all areas of each biopsy. The average count of 10 fields from each sample was recorded.

### Measurement of transcription factors for Treg/Th1/Th2/Th17

Samples used for transcription factor measurement were stored at −80°C until analysis. cDNA was synthesized from 2 μg of RNA with RevertAid™ H Minus Reverse Transcriptase (Thermo Fisher Scientific, Waltham, MA, USA), according to manufacturer's instructions. Levels of the transcription factors T-box transcription factor (T-bet); GATA binding protein 3 (GATA-3); retinoic acid-related orphan receptor (RORC); and, forkhead box P3 (FOXP3) were determined by real-time polymerase chain reaction (PCR). PCR reactions were performed using 1 μg cDNA (total RNA equivalent), 300 nmol/L primer pairs, and 1x SYBR Green Master Mix (Applied Biosystems, Foster City, CA, USA). Reactions were run using a StepOne™ Real-time PCR system (Applied Biosystems) for 40 cycles. Expressions of each gene were normalized to the mRNA of beta actin gene using StepOne™ software version 2.1 (Applied Biosystems). Data are shown as fold change in mRNA expression of the target gene in comparison to the beta actin mRNA level.

### Statistical analysis

Statistical analysis was performed using SPSS Statistics version 13.0 (SPSS, Inc., Chicago, IL, USA). Demographic and clinical data were interpreted using descriptive statistics. Data are reported as number and percentage, mean ± standard deviation, or median and interquartile range [IQR] (bar chart). Kruskal-Wallis test or Mann-Whitney U test (2-tailed) was employed for unpaired comparisons. Kruskal-Wallis test was used to compare between groups, and to determine intergroup variability. Mann-Whitney *U*-test was used for between-group comparisons. Baseline variables were analyzed by one-way analysis of variance (ANOVA) or by Fisher's exact test. Pearson's method was used for determining correlation coefficient. A *p* < 0.05 was regarded as being statistically significant.

## Results

### Patient characteristics

Forty-one patients were enrolled in this study. Mean age of patients was 45.93 ± 13.63 years, with a gender proportion breakdown of 22 males and 19 females. Twenty-six patients were diagnosed with CRScNP, seven with CRSsNP, and eight with CR. The eight patients diagnosed with CR comprised the control group in this study (Table 1).

**Table 1:** Demographic and clinical characteristics of patients (*N* = 41).

	**CRScNP**	**CRS**	**Controls**	***p*-value**
Patients (n)	26	7	8	
Gender (M:F)	13:13	3:4	6:2	
Age, mean ± SD	46.81 ± 12.71	46.29 ± 15.31	42.75 ± 16.39	0.521
Allergy, n (%)	13 (50.0%)	2 (28.6%)	5 (62.5%)	0.414
Aspirin Intolerance	none	none	none	
Asthma, n (%)	6 (23.1%)	0 (0%)	0 (0%)	0.049

*A p < 0.05 indicates statistical significance*.

*CRScNP, chronic rhinosinusitis with polyp; CRS, chronic rhinosinusitis; M, male; F, female; SD, standard deviation*.

### Histology

Of the 26 CRScNP specimens, six were grade I, nine were grade II, and 11 were grade III. The majority of CRScNP specimens (20/26, 76.9%) had eosinophil count greater than 100 cells/high-power field (HPF). Mean eosinophil count was 930.08 ± 1,399 cells/HPF (range 17–5,570; Tables 2, 3).

**Table 2:** Size of nasal polyps in the CRScNP group (*n* = 26).

**Polyp size**	**Patients**	**%**
I	6	23.1
II	9	34.6
III	11	42.3

*CRScNP, chronic rhinosinusitis with polyp*.

**Table 3:** Eosinophil count distribution among CRScNP patients (*n* = 26).

**Eosinophil count**	≤100	101–500	501–1,000	≥1001
Patients (n)	6	9	5	6
Percentage(%)	23.1	34.6	19.2	23.1

*CRScNP, chronic rhinosinusitis with polyp*.

### Transcription factor analysis

**Figure 1:**
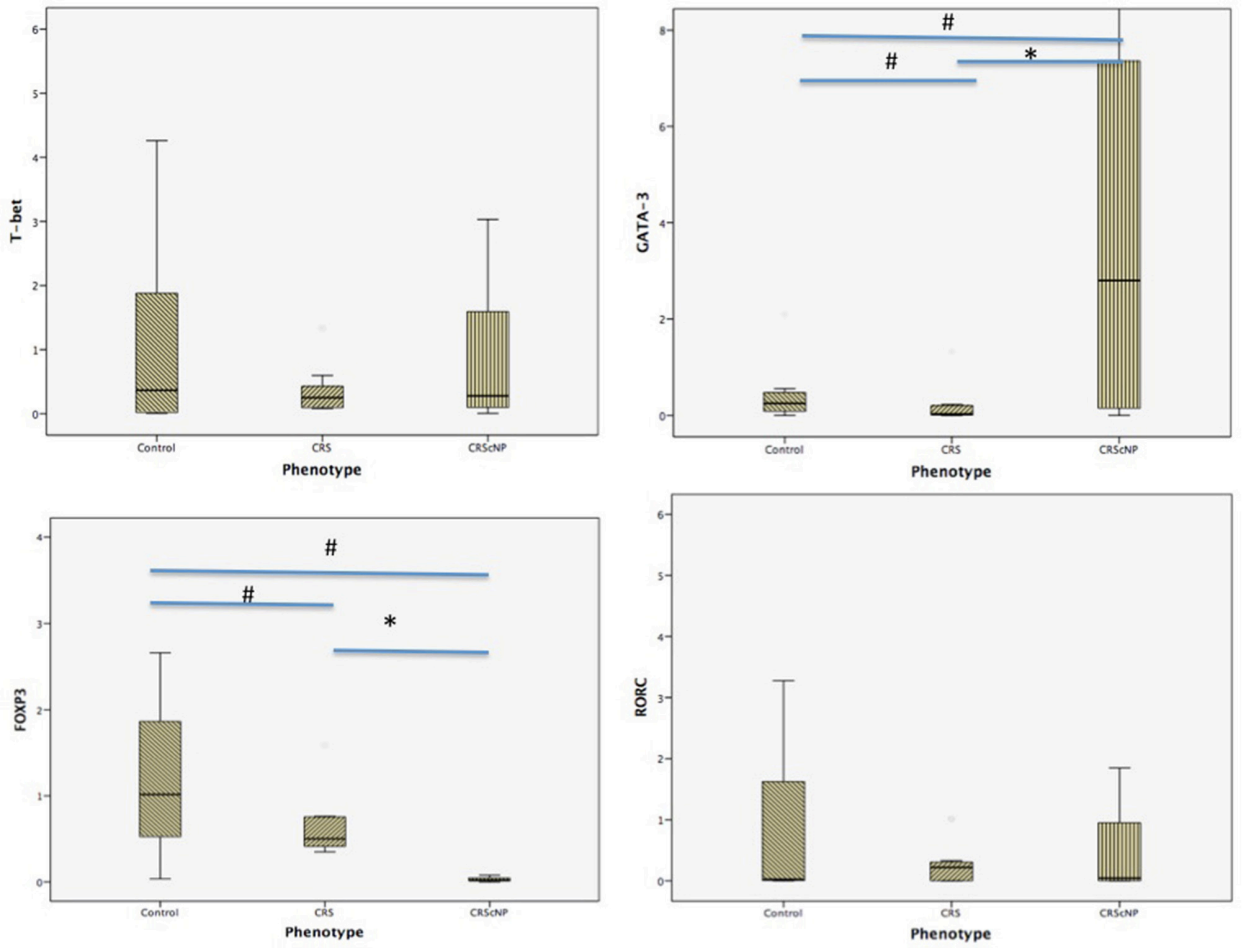
Comparison of each transcription factor among phenotypes (median [IQR]). CRScNP, chronic rhinosinusitis with polyp; CRS, chronic rhinosinusitis; T-bet, T-box transcription factor; GATA-3, GATA binding protein 3; FOXP3, forkhead box P3; RORC, retinoic acid-related orphan receptor C. ^*^Statistically significant between CRScNP and CRS ^#^Statistically significant comparing to Control group.

Th2 transcription factor (GATA-3) was statistically significantly higher in the CRScNP group than in the CRSsNP and control groups (*p* < 0.001); whereas, Treg transcription factor (FOXP3) was statistically significantly lower in the CRScNP group than in the CRSsNP and control groups (*p* < 0.001). The transcription factors for Th1 and Th17 (T-bet and RORC, respectively) were not significantly different among the three groups (*p* > 0.05; Table 4, Figure 1). The correlation between GATA-3 and eosinophilic count or allergic status was not statistically significant (Table 5).

**Table 4:** Transcription factor analysis of rhinosinusitis phenotypes.

**Transcription factor**	**CRScNP**	**CRS**	**Control**	***p*-value**
T-bet	7.64 ± 30.24	0.38 ± 0.45	1.09 ± 1.55	0.695
GATA-3	4.07 ± 4.11^*^^#^	0.25 ± 0.48^#^	0.46 ± 0.68	< 0.001
FOXP3	0.29 ± 0.02^*^^#^	0.68 ± 0.43^#^	2.41 ± 4.14	< 0.001
RORC	2.62 ± 7.47	0.26 ± 0.82	0.82 ± 1.47	0.847

*Data are reported as mean ± standard deviation*.

*A P < 0.05 indicates statistical significance*.

*CRScNP, chronic rhinosinusitis with polyp; CRS, chronic rhinosinusitis; T-bet, T-box transcription factor; GATA-3, GATA binding protein 3; FOXP3, forkhead box P3; RORC, retinoic acid-related orphan receptor C*.

* *Statistically significant between CRScNP and CRS*.

# *Statistically significant comparing to Control group*.

**Table 5:** Correlation between GATA-3 with eosinophilic count and allergic status.

	***p*-value**	**Correlation coefficient**
GATA3 with eosinophilic count	0.07	*r* = 0.23
GATA3 with allergic status	0.672	*r* = 0.068

*GATA-3, GATA binding protein 3*.

## Discussion

The main finding of this study was consistent with previously reported articles about polyps in Caucasians, which showed hyperfunction of Th2 and decreased activity of Treg (Zhang et al., 2008; Tomassen et al., 2016). Another important finding is the distribution of eosinophils in mucosa of CRScNP phenotype, which shifted from non-eosinophilic polyp to eosinophilic polyp. This shift was described in our previous report (Katotomichelakis et al., 2013), and in reports from Japan and Korea (Sakuma et al., 2011; Sejima et al., 2012).

There are different methods for evaluating and differentiating sinonasal inflammation. The most simplified method involves visual counting of eosinophils under light microscopy in HPF. Our first report of original data relating to the histologic profile of nasal polyp was published more than 10 years ago (Jareoncharsri et al., 2002). However, a better understanding of inflammatory pathways in pathogenesis is needed in the current era of precision/personalized medicine. TFA has been used to study lymphocytic activities and aberration of adaptive immunity. It requires less tissue sample comparing to the ELISA method. In this study, the relationship between Th2 transcription factor (GATA-3) and eosinophil count demonstrated a trend toward positive significant correlation; however, the association failed to achieve statistical significance. This finding suggests that the influx of eosinophils was not caused solely by Th2 hyperfunction.

An attempt to identify an eosinophil cut-off value in CRS with and without polyp was reported from two studies conducted in Japan (Sejima et al., 2012; Park et al., 2013). In the current study, we found the majority of polyps to have a large number of eosinophils. We also decided to analyze and report our data by grouping different eosinophil amounts/counts (Table 3) instead of using receiver operating characteristic (ROC) curves to determine the cut-off value.

The etiology of aberrant adaptive immunity remains largely unknown. An additional factor is defective innate immunity, most notably the mucosal defense mechanism. A hypothesis was recently proposed that centered on host-environment relationship and balance of microbiota (Harb and Renz, 2015; Toppila-Salmi et al., 2015). Unpublished data from our group relating to the results of bacterial culture between a group with allergic rhinitis group and a control group showed no difference between groups. A more sophisticated method of identifying bacterial organisms is needed to determine if there is a correlation between microbiota and mucosal inflammation.

This study has some mentionable limitations. First, the size of our study population was relatively small. As a result, our study may have lacked sufficient power of test (beta error) to identify all significant differences or associations. Second, we encountered a wide range of transcription factor values. Statistically significant difference among the three phenotypes was shown, but there was some overlap of upper range and lower range of confidence interval values between each phenotype. However, this finding successfully and importantly revealed shifts in the endotyping of sinonasal inflammation, which will facilitate the selection of appropriate therapeutic alternatives. Third and last, TFA is not a routine method for evaluating inflammation. As such, we are limited in our ability to extrapolate our findings into real-world clinical practice. We analyzed for correlation between eosinophil count and the quantitative measurement of Th2 and Treg function, and the result was no statistical significance (*p* = 0.07). Regardless, the main findings of Th2 hyperfunction and decreasing functional activity of Treg were consistent with other landmark studies.

## Conclusion

The result of transcription factor analysis revealed hyperfunction of Th2 in patients with CRScNP, which might result in hypereosinophilic infliltration in the polyps. One explanation for this finding is the decreased activity of Treg. Although environment-host interaction is the most probable hypothesis, the etiology of aberrant adaptive immunity needs to be elucidated.

## Author contributions

PT: designed experiments and study, recruited patients, collected samples, analyzed data, wrote and edited the manuscript; NS: analyzed the specimens, designed experiments; SM: collected samples and analyzed the specimens; JL, AB, and TS: recruited patients.

### Conflict of interest statement

The authors declare that the research was conducted in the absence of any commercial or financial relationships that could be construed as a potential conflict of interest. The reviewer AW and handling Editor declared their shared affiliation.
